# Prophylactic Mesh in Parastomal Hernia Prevention: Current Evidence

**DOI:** 10.3389/jaws.2025.15011

**Published:** 2025-07-30

**Authors:** Elisa Mäkäräinen

**Affiliations:** Gastrointestinal Surgery Department, Oulu University Hospital, Medical Research Center Oulu, Oulu, Finland

**Keywords:** parastomal hernia, prevention, keyhole technique, modified keyhole technique, Sugarbaker technique

## Abstract

**Introduction:**

Parastomal hernia (PSH) is a common long-term complication following stoma creation. The incidence of PSH exceeds 50% in long-term follow-up of end colostomy patients, while it remains lower in ileostomies and ileal conduit urinary diversions. PSH prevention strategies are of interest due to the poor outcomes and high recurrence rates associated with PSH repair.

**Overview of Techniques to Prevent PSH:**

Various technical approaches have been explored to reduce the risk of PSH. However, none have shown consistent benefit toward reducing PSH rate without the use of prophylactic mesh. The keyhole mesh technique was the first to demonstrate a significant reduction in PSH rates in early trials, but larger randomized controlled trials (RCTs) have later questioned its efficacy. The modified keyhole technique, using a funnel-shaped mesh, has shown promising results in recent small studies, with lower PSH incidence and potentially reduced stomal prolapse rate. Other methods such as the Sugarbaker technique and use of biological meshes in PSH prevention have been evaluated as well, with mixed results. While most research focuses on end colostomy, there is limited data on PSH prevention in ileostomies and ileal conduits.

**Conclusion:**

Despite early enthusiasm, the keyhole technique has not proven to be effective in preventing PSH. The modified funnel-shaped mesh appears to be a promising development, though long-term outcomes are lacking. Preventive mesh placement is still supported by international guidelines; however, these recommendations are not widely followed in colorectal surgery departments. Thus, further research is essential to guide future recommendations for PSH prevention.

## Introduction

Parastomal hernia (PSH) was first recognized as a complication of end colostomy in the 1990s [1]. PSH incidence is likely higher following end colostomy formation compared to ileostomy or ileal conduit urinary diversion [2]. In long-term follow-up studies, PSH rate has been shown to be over 50% for end colostomy patients [3, 4], while the incidence ranges from 7% to 36% for ileostomies [2, 5–7], and 10%–32% following ileal conduit urinary diversion [8–13].

PSH prevention is a reasonable goal due to high PSH incidence, especially after end colostomy without mesh, and the poor outcomes of PSH repair associated with high recurrence and complication rates [14]. PSH repair at the ileal conduit is seldom reported [15, 16]. Available data from small patient series indicate high morbidity and recurrence rates in line with findings from end colostomy PSH repairs [14–17].

## PSH Prevention Strategies

Initial prevention strategies focused on surgical techniques and the significance of right stoma placement for lowering PSH incidence [18, 19]. Various technical principles have been proposed to decrease the PSH rate. For example, the size of the fascial opening appears relevant. Therefore, excessively large openings should be avoided [4, 20]. Neither the transrectus nor pararectus positions have demonstrated superiority in PSH prevention [21–23]. Although extraperitoneal stoma creation may reduce the risk of parastomal hernia (PSH), robust evidence is lacking, as current data are based mainly on retrospective case series reporting relatively low PSH rates [24–27]. The opening technique (cruciate vs. circular) of the fascial orifice does not seem to have any impact on PSH risk [28]. In conclusion, PSH cannot be effectively prevented without a mesh.

## Keyhole Technique

The first randomized controlled trial (RCT) on PSH prevention used the keyhole mesh technique in the retrorectus space during open surgery [29]. Initial small-scale studies showed a significant reduction in PSH rates at 12 months’ follow-up [29, 30]. This method was widely adopted since, including in minimally invasive approaches. The keyhole technique has also been applied intraperitoneally with positive results during short-term follow-up [31]. However, larger recent RCTs have failed to confirm benefits of both retrorectus mesh placement [28, 32–34] and intraperitoneal keyhole technique [35], showing comparable PSH rates between mesh and non-mesh groups by both clinical and radiologic evaluation.

## Modified Keyhole Technique

To address shortcomings of the retrorectus keyhole technique in PSH prevention and repair, a funnel-shaped mesh was developed. Since the first publication of funnel-shaped mesh as PSH prevention in 2008 [36], over a dozen studies have supported its potential benefits without increased complications. Both case series [37, 38] and comparative observational studies [39–44] have shown significant reduction in PSH rates with mesh. Diagnostic methods varied between clinical and computer tomography (CT) -based evaluations. As a complication, stomal retraction was noted in 16.1% (5/31) of patients with funnel-shaped mesh [38]. One RCT of the funnel-shaped mesh has been published to date, reporting PSH rates of 2% in the mesh group vs. 43% in non-mesh group at 12-month clinical follow-up, and 10% vs. 37% by CT scan, respectively [45]. There has also been a trend toward reduced stomal prolapse in the mesh group [43, 45], though statistical significance has not been demonstrated.

## Other Techniques and Meshes

A single RCT has reported on the modified Sugarbaker technique, showing 22% PSH incidence in the mesh group compared to 44% in controls at 12 months’ follow-up [46]. A prospective case series using the same method reported a PSH rate of 7% [47]. Biological meshes are rarely used in PSH prevention [48, 49].

## Systematic Reviews and Meta-Analyses

Earlier systematic reviews and meta-analyses consistently supported prophylactic mesh use to prevent PSH [50–62] (Table 1). However, as recent RCTs have not confirmed the advantage of retrorectus keyhole mesh [28, 32–34], some reviews now advise against routine use of prophylactic mesh [63, 67]. When only studies using funnel shaped mesh were included, though, a significant reduction in the PSH rate was observed [68]. The European Hernia Society (EHS) continues to conditionally recommend use the prophylactic mesh with end colostomies [69]. However, if the patient is at high risk of developing a PSH, the recommendation is strong [69].

**TABLE 1:** Results of systematic reviews of parastomal hernia prevention with a mesh.

Author	Year	Stoma type	PSH (M-H, RR^1^/OR^2^, 95% CI)
Tam et al. [50]	2010	Colostomy	0.17 (0.07–0.40) All^2^
Wijeyekoon [51]	2010	Colostomy	0.23 (0.06–0.81) All^1^
Shabbir et al. [52]	2012	Colostomy	NR
Sajid et al. [53]	2012	Colostomy	0.11 (0.05–0.27) All^2^
Fortelny et al. [49]	2015	All	NR
			
Wang et al. [54]	2016	Colostomy	0.42 (0.22–0.82) All^1^
Zhu et al. [55]	2016	Colostomy	0.22 (0.13–0.38) All^1^
Chapman et al. [56]	2017	Colostomy, ileostomy	0.34 (0.18–0.65) All^1^
Cornille et al. [57]	2017	Colostomy, ileostomy	0.40 (0.21–0.75) All^1^ 0.36 (0.17–0.77) Synthetic mesh^1^ 0.58 (0.11–2.95) Biological mesh^1^
Cross et al. [58]	2017	Colostomy, ileostomy	0.24 (0.12–0.50) All^2^
Lopez-Cano et al. [59]	2017	Colostomy	0.43 (0.26–0.71) All^1^ 0.30 (0.15–0.59) Retrorectus mesh^1^ 0.62 (0.36–1.07) Intra-abdominal^1^
Patel et al [60]	2017	Colostomy	0.21 (0.11–0.38) All^2^
Pianka et al. [61]	2017	Colostomy, ileostomy	0.24 (0.10–0.58) All^2^
Jones et al. [62]	2018	Colostomy	0.53 (0.43–0.66) All^1^ 0.48 (0.36–0.64) Retrorectus mesh^1^ 0.76 (0.55–1.06) Intra-abdominal mesh^1^
Prudhomme et al. [63]	2021	Colostomy	0.73 (0.51–1.07) All^1^ 0.76 (0.43–1.34) Retrorectus mesh^1^ 0.66 (0.36–1.22) Intra-abdominal mesh^1^
Sahebally et al. [64]	2021	Colostomy	0.27 (0.12–0.61) All^2^
Dewulf et al. [65]	2022	Ileal conduit	NR
Hinojosa-Gonzalez et al. [66]	2024	Colostomy, ileostomy, ileal conduit	0.52 (035–0.77) Colostomy^2^ 0.49 (0.25–0.97) Ileal Conduit^2^ NR Ileostomy
Verdaguer-Tremolosa et al. [67]	2024	Colostomy	0.68 (0.46–1.02) All^2^
Pompeu et al. [68]	2025	Colostomy	0.07 (0.03–0.17) Funnel shaped mesh^2^

1 RR risk ratio.

2 OR odds ratio.

PSH, parastomal hernia; CI, Confidence Interval; M-H, Mantel-Haenszel method.

## Ileostomy PSH Prevention

PSH prevention in ileostomies is less frequently studied, partly due to lower incidence [2, 5–7] and possibly due to frequent association of ileostomies with inflammatory bowel disease, which may have led to reluctance to use mesh. In a small cohort publication, retrorectus keyhole mesh failed to prevent PSH [70], as did biological mesh [48] in ileostomies.

## Ileal Conduit PSH Prevention

Ileal conduit PSH prevention and repair remains poorly studied topics. The PSH incidence is estimated at 10%–32% [8–13] but can be as high as 68% by CT scan [7]. Most cases are asymptomatic and do not require surgical repair. Thus far, keyhole mesh techniques have been trialed in ileal conduit procedures for PSH prevention. Liedberg et al. [71] reported a significant reduction in PSH using retrorectus keyhole mesh. However, Donahue et al. found an 18% PSH rate on CT after a median follow-up of 297 days, despite the retrorectus keyhole mesh [72]. A recent RCT using intra-abdominal biological keyhole mesh found no benefit in PSH prevention [73].

## Discussion

The era of PSH prevention began two decades ago with promising results using the keyhole technique. However, its efficacy in end colostomy PSH prevention has since been questioned in large RCTs with no difference in PSH rate between the mesh and non-mesh groups. The modified keyhole technique appears to offer a potential alternative with encouraging results published so far. Until 2016, only funnel-shaped mesh with a 2-cm-long funnel was available. Studies have widely used this version of mesh [41–43]. Today, the mesh with 4-cm long funnel is commonly used to provide better coverage around the bowel, therefore possibly decreasing the likelihood of PSH [45].

As most studies focus on end colostomies, EHS and The Association of Coloproctology of Great Britain and Ireland (ACPGBI) recommend prophylactic mesh in permanent colostoma formation only [4, 69, 74]. Recommendations on ileostoma or ileal conduit parastomal hernia prevention cannot be given, despite the poor results of repair and relatively high PSH rate [2, 5–7, 15]. Despite the recommendations, mesh use remains limited [75, 76]. Additionally, the intra-abdominal location of modified keyhole meshes and lack of long-term follow-up results may hinder wider adoption, although no significant mesh-related complications have been reported due to the location of the mesh.

Ileal conduit PSH prevention may follow a similar progression to end colostoma PSH prevention. By far, retrorectus keyhole mesh has shown benefits in ileal conduit PSH rate. However, further research is needed to trial especially the modified keyhole technique in both ileal conduits and end ileostomies as well. Additionally, in modified keyhole technique studies the patient populations have had relatively low mean BMIs and further research should focus on mesh usability in higher BMI patient populations.

## Conclusion

Parastomal hernia is still a common and challenging complication after stoma creation even two decades after the introduction of PSH prevention techniques. While the traditional keyhole mesh technique does not prevent PSHs, the modified funnel-shaped mesh offers a potential option. Existing data, although limited, suggest reduction in PSH incidence without increased complications. However, further high-quality, large-scale research, particularly in patients with higher BMI and in ileal conduit or ileostomy populations with sufficient follow-up time, is needed to guide future practice. Despite current guidelines and recommendations, prophylactic mesh remains underutilized.
